# Practical Approach to Transcatheter Aortic Valve Implantation and Bioprosthetic Valve Fracture in a Failed Bioprosthetic Surgical Valve

**DOI:** 10.1155/2022/9899235

**Published:** 2022-02-15

**Authors:** Maarten Vanhaverbeke, Ole De Backer, Christophe Dubois

**Affiliations:** ^1^The Heart Centre, Rigshospitalet, Copenhagen, Denmark; ^2^Department of Cardiovascular Medicine, University Hospitals Leuven, Leuven, Belgium; ^3^Department of Cardiovascular Sciences, KU Leuven, Leuven, Belgium

## Abstract

Bioprosthetic surgical aortic valve failure requiring reintervention is a frequent clinical problem with event rates up to 20% at 10 years after surgery. Transcatheter aortic valve-in-valve implantation (ViV-TAVI) has become a valuable treatment option for these patients, although it requires careful procedural planning. We here describe and illustrate a stepwise approach to plan and perform ViV-TAVI and discuss preprocedural computerized tomography planning, transcatheter heart valve selection, and implantation techniques. Particular attention is paid to coronary artery protection and the possible need for bioprosthetic valve fracture since patients with small surgical aortic bioprostheses are at a risk of high residual gradients after ViV-TAVI. Considering updated clinical data on long-term outcomes following ViV-TAVI, this approach may become the default treatment strategy for patients with a failing surgical aortic bioprosthesis.

## 1. Introduction

Surgical implantation of a bioprosthetic aortic valve (aortic valve replacement, AVR) has been the treatment of choice for many patients with aortic valve stenosis (AS) or regurgitation. Current guidelines of the ACC/AHA (American College of Cardiology/American Heart Association) and ESC/EACTS (European Society of Cardiology/European Association for Cardio-Thoracic Surgery) recommend surgical AVR for patients below, respectively, 65 or 75 years of age, who have symptomatic AS or severe asymptomatic AS, provided they have a long life expectancy [1, 2]. However, bioprosthetic aortic valve failure is frequent, with event rates ranging from 5% up to 20% at 10-year follow-up, depending on the valve type [3, 4]. Bioprosthetic valve failure is defined as prosthetic dysfunction which leads to valve-related death, repeat intervention, or severe hemodynamic structural valve degeneration (a mean gradient of 40 mmHg or more or a 20 mmHg increase compared to immediately after implantation) [5].

In the recently updated ESC guidelines (2021), redo cardiac surgery is a class I, level of evidence C indication for symptomatic patients with bioprosthetic valve failure (after excluding thrombosis and endocarditis) and class IIa (C) indication for asymptomatic patients with low surgical risk [1]. However, transcatheter aortic valve-in-valve implantation (ViV-TAVI) has gained much attention in recent years because of the high procedural success rates of more than 90%. ViV-TAVI has been upgraded from a class IIa (C) in the 2017 ESC guidelines to class IIa (B) recommendation in the 2021 ESC guidelines and should be considered based on anatomical characteristics and features of the surgical prosthesis and in patients at high surgical risk. These recommendations are based on registry data and propensity-matched registry studies, showing better short- and long-term outcomes with ViV-TAVI vs. redo surgery [6–8]. Considering these data and new insights, ViV-TAVI may become the preferred treatment for bioprosthetic valve failure, irrespective of the surgical risk category of the patient.

However, ViV-TAVI requires meticulous planning of the procedure and selection of the approach to minimize the risk of coronary obstruction, device malposition, and high residual gradients [9]. While multiple studies have shown that patients with bioprosthetic valves with small dimensions (21 mm or lower) or with high gradients (patient-prosthesis mismatch, PPM) are at an increased risk of early degeneration, these patients are also at risk for high residual gradients after ViV-TAVI [10].

**Figure 1:**
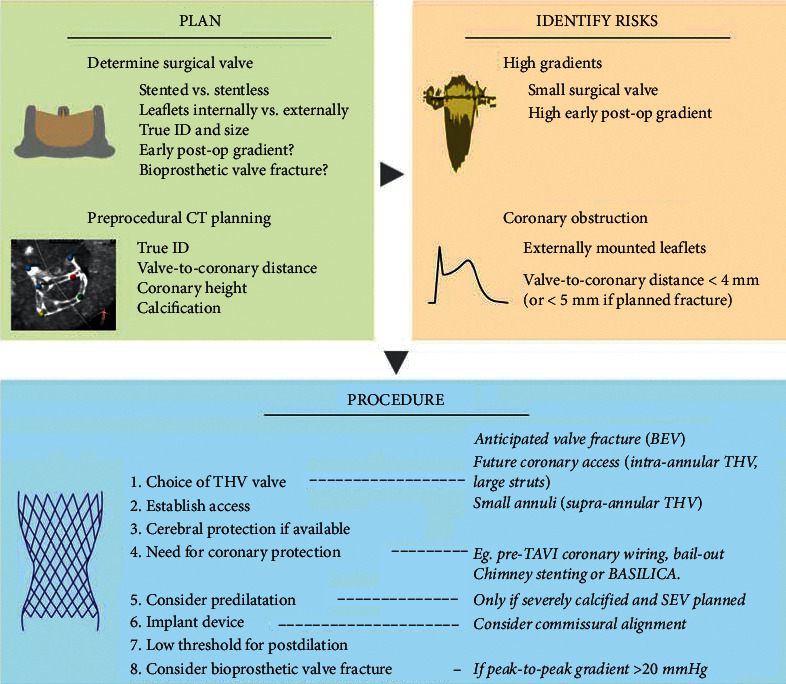
Practical approach to ViV-TAVI, with preprocedural planning to identify and address coronary occlusion or high residual gradients after TAVI. BEV: balloon-expandable valve; SEV: self-expandable valve; THV: transcatheter heart valve; TAVI: transcatheter aortic valve implantation; ID: internal diameter; BASILICA: bioprosthetic aortic scallop intentional laceration to prevent iatrogenic coronary artery obstruction.

We here systematically describe the approach to plan and perform a ViV-TAVI (Figure 1), carefully addressing the issues mentioned above, with particular attention to coronary artery protection strategies and bioprosthetic valve fracture (BVF) to avoid high residual gradients in case of small aortic bioprostheses.

## 2. Surgical Aortic Bioprosthetic Valve Types

When assessing a potential candidate for ViV-TAVI, the first step is to identify the type and size of the surgical bioprosthesis as this may identify procedural risks (e.g., coronary occlusion because of externally mounted leaflets) and influence the approach (e.g., bioprosthetic valve fracture). Surgical bioprostheses can be classified into stented, stentless, or sutureless valves (Table 1). Stented valves may have internally mounted leaflets, resulting in a true inner diameter (ID) that is smaller than the labeled valve size. To maximize effective orifice area (EOA), surgical bioprostheses with externally mounted leaflets (e.g., St. Jude Trifecta and Sorin Mitroflow) and stentless valves have been designed.

In the Valve-in-Valve International Data (VIVID) registry, reporting on the outcomes in 1600 ViV-TAVI procedures, the main challenge in stented valves was reported to be a high residual gradient or PPM [12]. In contrast, stentless valves are more challenging because of the lack of fluoroscopic markers, an increased risk of device malposition (10.3% versus 6.2% in stented valves, *p*=0.014), coronary obstruction (6.0% vs. 1.5%, *p* < 0.001), and paravalvular leak (PVL, 11% vs. 4.5%, *p* < 0.001). Coronary obstruction following ViV-TAVI is also a major concern in patients with stented valves with externally mounted leaflets [13].

**Figure 2:**
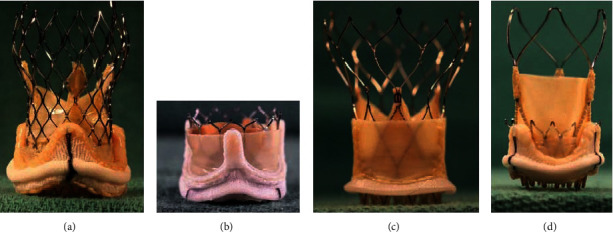
Ex vivo examples of THVs mounted in surgical bioprostheses. (a) Evolut R 23 mm THV in an Epic 21 mm surgical valve. (b) Sapien 23 mm THV mounted in a Perimount Magna Ease 21 mm surgical valve. (c) Portico 23 mm THV mounted in a Trifecta 21 mm valve with externally mounted leaflets. (d) ACURATE neo S mounted in Epic 25 mm. Images were obtained from the valve-in-valve aortic app (by UBQO and Dr. Vinayak Bapat), reproduced with permission.

Once the surgical valve has been identified, the “valve-in-valve aortic” app (by UBQO and Dr. Vinayak Bapat) can be used to identify which THV types can be used for ViV-TAVI. The aortic ViV app also provides information on the true internal diameter, fluoroscopic landmarks to correctly position the THV, and the possibility of valve fracturing. Ex vivo images of the transcatheter heart valves (THVs) mounted inside surgical bioprostheses, obtained from the aortic ViV app, are shown in Figure 2 (internally mounted leaflets in Figures 2(a), 2(b), and 2(d) and externally mounted leaflets in Figure 2(c)).

## 3. Preprocedural Computerized Tomography Planning

**Figure 3:**
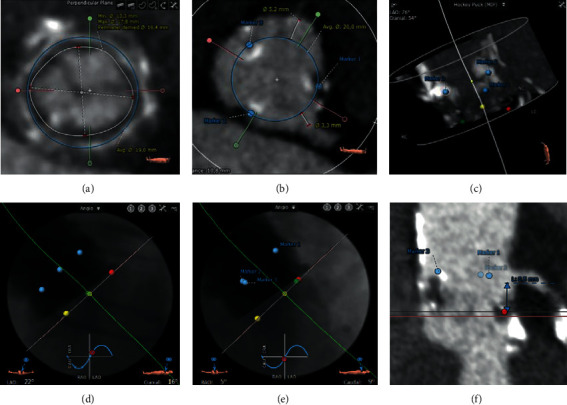
Preprocedural CT planning in a 91-year-old female patient with high-grade aortic stenosis (peak gradient 64 mmHg and mean gradient 35 mmHg) that underwent ViV-TAVI in a failed 21 mm Epic bioprosthesis. (a) The basal ring of the Epic valve is marked at the center of the cusps (red, blue, and green dots). The stent diameter is 19 mm, and true internal diameter is 16.4 mm (17 mm according to the aortic ViV app, with internally mounted leaflets). (b) The posts of the stent of the Epic valve are marked with generic markers (blue). The blue circle is centered on the surgical valve and represents the waist of the planned 23 mm Evolut valve. The distance between the virtual 23 mm valve (with the waist of 20 mm) and the left coronary (virtual transcatheter valve-to-coronary ostium distance, VTC) is low (3.3 mm). (c) Hockey puck view showing the generic blue markers on the top posts, while the colored markers are in the middle of the cusps at the base of the surgical valve. (d) Coplanar view with the left coronary cusp at the right side (red marker). (e) Cusp-overlap view with overlapping left and right coronary cusps (red and green markers). (f) Low left coronary height (8.5 mm) with shallow sinuses of Valsalva.

Preprocedural computerized tomography (CT) planning is the key when performing ViV-TAVI (Figure 3). The stent diameter of the valve and true ID can be measured and should be in line with the expected values based on the surgical valve type. In case of internally mounted leaflets, the true ID can be smaller than the stent diameter. The upper stent posts of the surgical valve can be marked with three generic markers which can then be used to determine the optimal fluoroscopic views (i.e., three-cusp coplanar view and left/right coronary (LCC/RCC) cusp-overlap).

Next, it is important to identify anatomical factors which may lead to coronary obstruction. When implanting a THV in a surgical bioprosthesis, the leaflets are displaced outward and may occlude the coronary ostia. The implanted THV is not necessarily restricted by the surgical valve, especially not at the level of the coronary ostia since the surgical valve commissures are most often aligned with the native valve [13, 14]. This is also the reason why surgical bioprostheses with externally mounted leaflets or stentless valves have a higher risk of coronary occlusion. The sinuses can even be completely sealed off when the displaced leaflets extend to the sinotubular junction (sinus sequestration), which is more likely in the case of a narrow sinotubular junction or TAVI-in-TAVI [15]. The risk for coronary obstruction can be assessed by measuring the virtual transcatheter valve-to-coronary (VTC) ostium distance and the valve-to-sinotubular junction distance. The VTC distance is measured from the ostium of the coronaries to a virtual cylinder, aligned at the base of the surgical valve, extending up to the coronary ostia and with a diameter equal to the planned THV or its waist at that level (Figure 3). It has been shown that patients with a VTC distance ≤4 mm are at an increased risk of coronary obstruction, and a cutoff of ≤3 mm is considered high risk [13, 14].

## 4. Choice of the Transcatheter Heart Valve

The US Food and Drug Administration (FDA) has approved the balloon-expandable (BEV) Sapien XT and Sapien 3 (Ultra) valve (Edwards Lifesciences) with intra-annular leaflet position and the self‐expanding (SEV) Evolut platform (Medtronic) with supra-annular leaflet position for ViV-TAVI. Other platforms can be used off-label (Figure 2). Differences in valve stent frame design, leaflet position, and THV expansion/implantation may potentially be beneficial in specific situations.

To prevent high gradients after ViV-TAVI, a THV with supra-annular leaflet position may theoretically be preferred to maximize the EOA. Especially, patients with small annuli, stented bioprostheses, and a small EOA are at risk for high transvalvular gradients after ViV-TAVI. In the VIVID registry, the use of the Sapien device was an independent predictor for increased gradient after ViV-TAVI (mean gradient >20 mmHg, odds ratio: 2.3) [9]. These higher postprocedural gradients with Sapien were especially observed in patients with small annuli (<20 mm) (43% for Sapien versus 24% for the CoreValve/Evolut platform) [16]. No significant difference in postprocedural gradients was reported for patients with larger annuli (23 mm or more), with rates of 21% in both groups. Importantly, at longer-term follow-up, the use of Sapien was associated with higher reintervention rates, caused by higher postprocedural gradients [17].

While a SEV with supra-annular leaflet position may be preferred to maximize the EOA, device malposition is more common when using the SEV as compared to BEV [12]. However, with increasing operator experience and the introduction of repositionable SEV, the rates of device malposition have markedly dropped from 15% in 2012 to 6.5%, and this number can be expected to further decrease [9, 17]. The reported rates of PVL have also been higher for the SEV as compared to BEV, although again, the rate of PVL was reported to be reduced with newer generation devices [12]. No difference in all-cause mortality has been reported for BEV versus SEV in ViV-TAVI procedures [16].

Finally, the need for future coronary access and the need for a possible future re-ViV-TAVI can also guide the THV type selection [18]. A lower stent frame or intra-annular design (Sapien platform) or bigger stent struts are preferred when the possibility of future coronary access needs to be optimized. An intra-annular valve design with low stent height (Sapien platform) may also facilitate future re-ViV-TAVI.

## 5. Implantation Techniques

**Figure 4:**
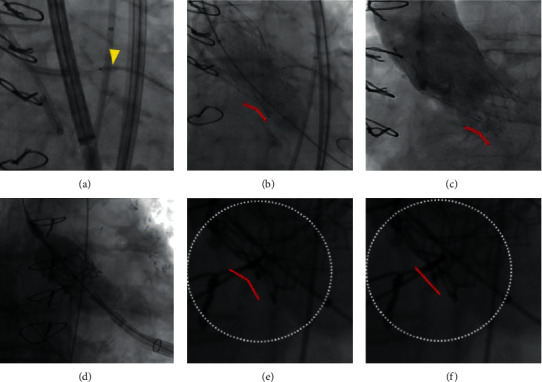
ViV-TAVI with bioprosthetic valve fracture and suboptimal result in a transfemoral (a–c) and good result in a transapical (d–f) approach. (a) In the 91-year-old female patient from Figure 3, because of low coronary height, anticipated bioprosthetic valve fracture, and shallow sinuses, coronary protection was obtained using a 6 F guiding catheter, 6 F GuideLiner, and undeployed stent standby (yellow arrowhead). A 23 mm Evolut R valve was implanted 4 mm under the fluoroscopic ring of the Epic valve as a reference. (b) Invasive gradient after valve implantation was 26 mmHg. BVF was attempted using an 18 mm True balloon at 14 atm. (c) No change in surgical ring geometry or pressure drop on the indeflator was noticed, but the invasive gradient at the end of the procedure was only 3 mmHg. However, the noninvasive peak transvalvular gradient increased to 60 mmHg at 3 months after TAVI, and the patient was rehospitalized with heart failure. Potentially, the 18 mm balloon (true ID + 1 mm) was slightly undersized to achieve fracturing. (d) In contrast, an 85-year-old patient with extensive peripheral vascular disease and a degenerated Perimount Magna Ease 21 mm valve (true ID: 19 mm) underwent transapical implantation of a 23 mm Sapien 3 valve. Postimplantation invasive gradient was 25 mmHg. (e) Postdilatation with Atlas Gold 22 mm balloon. (f) Sudden geometric expansion of the valve at the end of the inflation and a pressure drop on the indeflator, with a reduction of the gradient to 9 mmHg (circles in (e) and (f) denote the similar region in video 1).

In contrast to native valve TAVI, the THV in ViV-TAVI is positioned relative to the fluoroscopic landmarks of the surgical aortic bioprosthesis (usually 2–4 mm below the surgical valve) and not to the annular plane (Figure 4(a)). The optimal implantation depth is also provided by the aortic ViV app. A radiopaque ring at the inflow part of the surgical aortic bioprosthesis may facilitate this, while it is more difficult when there are only radiopaque markers of the surgical valve posts (e.g., Mosaic) or no radiopaque markers at all (e.g., stentless valves). When severe calcification is observed on the preprocedural CT scan, predilatation should be considered to avoid severe underexpansion of a self-expanding THV. However, there is no indication for routine predilatation. Patient-specific commissural alignment can be obtained by aligning the THV with the surgical valve, using the predefined fluoroscopic views and generic markers outlined above [19]. For Evolut, the hat marker should be at the center front during implantation, and the C-tab should be on the inner curve after valve deployment in the R/L cusp-overlap view. No implantation techniques are currently available to provide patient-specific commissural alignment when using Sapien, although this is less critical because of the lower profile of the valve. Standard pacing approaches are used during implantation of the BEV, i.e., 180/min or up to 200–220/min if slower rates do not provide enough pressure decrease. In case of implantation of the SEV in surgical aortic bioprostheses, it is typically best to use intermediate rate pacing, starting at 120–130/min but often transiently increasing the pacing rate to 160/min for a few seconds during valve expansion. Often, postdilatation is performed to optimize the hemodynamic result. The risk of inducing conduction disorders and permanent pacemaker implantation is low (typically <5%) [16]. For Evolut valves, it is recommended to size the postdilatation balloon to the true ID of the surgical valve or 1 mm smaller. For the Sapien platform, the valve is usually oversized minimally 1 mm based on the true ID [20].

## 6. Bioprosthetic Valve Fracture

Routine postdilatation of the SEV should be considered as high residual transvalvular gradients have consistently been shown to be associated with increased mortality [17, 21]. When residual invasive peak-to-peak gradients of 20 mmHg or more are measured after the ViV-TAVI procedure, bioprosthetic valve fracture (BVF) can be considered (Figures 4(b)–4(f)). BVF will be more likely necessary if the immediate postoperative gradients after SAVR were already high (PPM). BVF also reduces pinwheeling, especially in the case of Sapien valves. While this maneuver may be considered to be relatively aggressive, BVF was shown to be safe in a multicenter registry [22]. Whether BVF should be performed just before or after the THV implantation is still a matter of debate among experts; solid real-world data on this topic are still missing.

An indication on which surgical valves can be fractured is shown in Table 1. In general, sutureless and stentless valves cannot be fractured, but they can potentially be overexpanded. To perform BVF, a 50 cc syringe is used, connected with a 3-way stopcock to a noncompliant (NC) high-pressure balloon (True or Atlas Gold balloon (Becton, Dickinson and Company, New Jersey, US)) and an indeflator. It is recommended to fill the 50 cc syringe and indeflator with a 20% contrast-80% saline mixture. After positioning the balloon at the level of the valve, rapid pacing (180–220/min) is initiated. The balloon is quickly inflated with the 50 cc syringe (volume phase, which takes 3–5 seconds) after which the 3-way stopcock is opened to the indeflator to allow pressures up to 14 atm (pressure phase, which can take up to 15–20 seconds). Fracturing of the frame is confirmed visually on fluoroscopy or when a pressure drop is observed on the indeflator (Figures 4(e) and 4(f) and video 1). The indeflator is then released, the balloon is deflated, and the invasive gradients can be measured again.

Typically, the size of the balloon is 1 mm larger than the labeled valve size or 1–3 mm larger than the true ID [11, 23, 24]. Postdilatation balloon sizes have to be carefully chosen in case of the Evolut valve to avoid trauma to the Evolut leaflets. Medtronic recommends that the NC postdilatation balloon does not exceed 1 mm more than the THV waist diameter (i.e., a 20 mm waist in a 23 mm Evolut valve). Intraventricular balloon positioning can be performed if postdilatation with a larger NC balloon is needed. BVF can increase the surgical frame diameter by 2–4 mm. As a consequence, it is also reasonable to use a safety margin when measuring the VTC distance (e.g., 5 mm instead of 3-4 mm).

Achieving a good hemodynamic result after BVF is important since a high residual gradient results in a poor clinical outcome, as illustrated in the two examples in Figure 4. Nevertheless, it can often be difficult to observe geometric changes in the stent frame. Moreover, there can be a discrepancy between invasively measured gradients after BVF and the final result measured noninvasively days to weeks after the implantation [25].

## 7. Coronary Protection Strategies

Coronary obstruction is a rare but devastating complication of ViV-TAVI, with rates of approximately 3% [13]. Meticulous preprocedural cardiac CT analysis can identify risk factors for coronary obstruction, as previously discussed. In a multivariable model, VTC distance and the use of stented bioprostheses with externally mounted leaflets or stentless bioprostheses were independent risk factors for coronary obstruction [13]. BVF may also increase the risk of coronary obstruction. To mitigate this risk, a THV with a lower stent frame height may sometimes be preferred.

**Figure 5:**
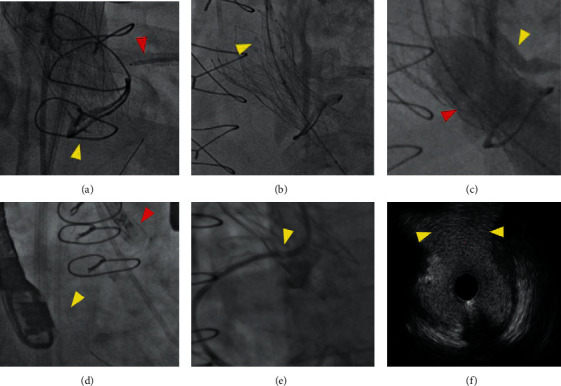
Coronary protection using chimney stenting (a–c) or BASILICA (d, e). (a) An 83-year-old male patient underwent transfemoral ViV-TAVI because of severe aortic regurgitation in a bioprosthetic valve of unknown type. Because of low coronary height, shallow sinuses, VTC 1 mm, and advanced age, coronary protection was obtained with a wire, 6 F GuideLiner, and unexpanded stent in the LAD (red arrowhead). Device malposition of a 25 mm Navitor valve occurred, resulting in severe aortic regurgitation (yellow arrowhead). (b) A second Navitor 25 mm valve was implanted with a good result (double layer of markers of the Navitor valve, yellow arrowhead). (c) Chimney stenting was performed using a Synergy Megatron 4.0 × 20 mm DES with kissing balloon inflation with 6.0 × 20 mm Emerge NC (yellow arrowhead) and 22 mm True balloon (red arrowhead). (d) A 63-year-old female patient underwent transfemoral ViV-TAVI in a 21 mm Trifecta valve. Because of low right coronary ostium (10 mm), VTC of 3 mm, shallow sinuses, and externally mounted leaflets, the right coronary was protected with a wire (yellow arrowhead) and 6 F GuideLiner (temporarily retracted in the 6 F guiding catheter). Because of the young age, a BASILICA procedure was performed upfront (red arrowhead, set up before leaflet laceration with an 8 F traversal MP1 guiding catheter with Astato XS 20 wire inside the PiggyBack wire converter and snared Astato wire inside a 6 F MP guiding catheter). (e) Haziness at the right coronary ostium after successful implantation of a Navitor 23 mm valve 4 mm below the fluoroscopic ring. (f) IVUS showing a nice opening at the level of the right coronary ostium towards the aorta (yellow arrowheads) BASILICA: bioprosthetic aortic scallop intentional laceration to prevent iatrogenic coronary artery obstruction; IVUS: intracoronary vascular ultrasound; DES: drug-eluting stent; VTC: virtual transcatheter to coronary distance.

In elderly patients at the risk of coronary obstruction, protection with a coronary guidewire, with or without undeployed stent standby in the coronary artery, is a reasonable strategy. In case of obstruction or anticipated very high risk of obstruction (e.g., Figures 5(a)–5(c), bail-out TAVI-in-TAVI in a patient already at a high risk of coronary obstruction), chimney stenting can be performed. In younger patients, in whom the need for future coronary access is important, a preemptive intervention to safeguard coronary access should be considered. In the recently described BASILICA technique (Bioprosthetic Aortic Scallop Intentional Laceration to prevent Iatrogenic Coronary Artery obstruction), the bioprosthetic leaflet is punctured from the side of the sinus, snaring a wire in the left ventricular outflow tract, externalizing it, and then lacerating the leaflet using electrocauterization (Figure 5(d)) [26–29]. By lacerating the leaflet and creating a V-shaped surgical valve leaflet, the risk of coronary occlusion by the bioprosthetic leaflet is reduced when it is pushed to the side by the THV. Although there is a substantial learning curve, the safety and feasibility of this technique have been documented in a prospective cohort study [30]. To assess whether the coronary ostium is patent at the end of the ViV-TAVI procedure, an aortogram in an isolated RCC or LCC cusp view can be performed. In case of doubt, IVUS can also be helpful (Figures 5(e) and 5(f)).

## 8. Cerebral Protection

**Figure 6:**
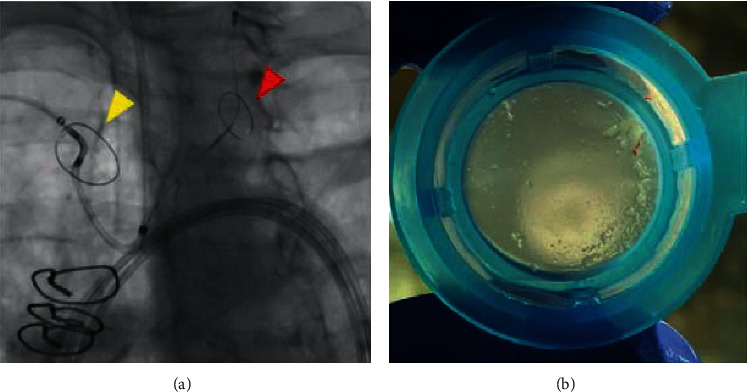
Cerebral protection in ViV-TAVI. (a) Sentinel^®^ cerebral protection device, implanted with filters in the brachiocephalic trunk and left common carotid artery. (b) Filter from the Sentinel cerebral protection device from a valve-in-valve case showing debris.

Ischemic stroke risk after ViV-TAVI has been reported to be approximately 2%, but no information on the use of cerebral protection is available in these reports [12, 17]. As the risk for embolic stroke or debris may theoretically be higher in ViV-TAVI, it may be advisable to use cerebral protection during these procedures, especially when performing BASILICA-assisted ViV-TAVI (Figure 6).

## 9. Conclusion

ViV-TAVI is a safe and valuable treatment option to treat failed surgical aortic bioprostheses, provided that the procedure is carefully planned and performed. Besides newer THV generations with better implantation results, also newer techniques for coronary protection and cerebral protection are now available to mitigate the major risks of ViV-TAVI. Achieving good hemodynamic results with low gradients after implantation is the key to ascertain good long-term outcome.

## Figures and Tables

**Table 1:** Design, characteristics, and challenges of commonly used surgical aortic valves.

	Leaflets mounted	Fracture potential [11]	Challenges
Stented valves			(i) Higher postprocedural gradients (ii) Coronary obstruction (externally mounted leaflets)
Sorin Mitroflow	Externally	Yes (19–21 mm)	
St. Jude Trifecta	Externally	No	
St. Jude Biocor Epic	Internally	Yes (21 mm)	
Medtronic Mosaic	Internally	Yes (19–21 mm)	
Medtronic Hancock II	Internally	No	
Edwards Magna Ease	Internally	Yes (19–21 mm)	
Edwards Magna	Internally	Yes (19–21 mm)	
Edwards Perimount 2700	Internally	No, but expandable	
Edwards Perimount 2800	Internally	Yes	
Labcor Porcine	Internally	Yes	

Stentless valves			(i) Lack of fluoroscopic markers (ii) Device malposition (iii) Coronary obstruction (iv) Paravalvular leak
Sorin Freedom			
St. Jude Toronto			
Medtronic Freestyle	Extended full porcine root		
Edwards Prima Plus	Extended full porcine root		

Sutureless			
Sorin Perceval	Internally		
Edwards Intuity	Internally		
Medtronic Enable	Internally		

## References

[1] Vahanian A., Beyersdorf F., Praz F. (2021). 2021 ESC/EACTS Guidelines for the management of valvular heart disease. *European Heart Journal*.

[2] Otto C. M., Nishimura R. A., Bonow R. O. (2021). 2020 ACC/AHA guideline for the management of patients with valvular heart disease: a report of the American College of Cardiology/American heart association joint committee on clinical practice guidelines. *Circulation*.

[3] Rodriguez-Gabella T., Voisine P., Puri R., Pibarot P., Rodés-Cabau J. (2017). Aortic bioprosthetic valve durability. *Journal of the American College of Cardiology*.

[4] Hammermeister K., Sethi G. K., Henderson W. G., Grover F. L., Oprian C., Rahimtoola S. H. (2000). Outcomes 15 years after valve replacement with a mechanical versus a bioprosthetic valve: final report of the Veterans affairs randomized trial. *Journal of the American College of Cardiology*.

[5] Capodanno D., Petronio A. S., Prendergast B. (2017). Standardized definitions of structural deterioration and valve failure in assessing long-term durability of transcatheter and surgical aortic bioprosthetic valves: a consensus statement from the European Association of Percutaneous Cardiovascular Interventions (EAPCI) endorsed by the European Society of Cardiology (ESC) and the European Association for Cardio-Thoracic Surgery (EACTS). *European Journal of Cardio-Thoracic Surgery*.

[6] Deharo P., Bisson A., Herbert J. (2020). Transcatheter valve-in-valve aortic valve replacement as an alternative to surgical re-replacement. *Journal of the American College of Cardiology*.

[7] Tam D. Y., Dharma C., Rocha R. V. (2020). Transcatheter ViV versus redo surgical AVR for the management of failed biological prosthesis: early and late outcomes in a propensity-matched cohort. *JACC: Cardiovascular Interventions*.

[8] Hirji S. A., Percy E. D., Zogg C. K. (2020). Comparison of in-hospital outcomes and readmissions for valve-in-valve transcatheter aortic valve replacement vs. reoperative surgical aortic valve replacement: a contemporary assessment of real-world outcomes. *European Heart Journal*.

[9] Dvir D., Webb J., Brecker S. (2012). Transcatheter aortic valve replacement for degenerative bioprosthetic surgical valves: results from the global valve-in-valve registry. *Circulation*.

[10] Flameng W., Herregods M.-C., Vercalsteren M., Herijgers P., Bogaerts K., Meuris B. (2010). Prosthesis-patient mismatch predicts structural valve degeneration in bioprosthetic heart valves. *Circulation*.

[11] Allen K. B., Chhatriwalla A. K., Cohen D. J. (2017). Bioprosthetic valve fracture to facilitate transcatheter valve-in-valve implantation. *The Annals of Thoracic Surgery*.

[12] Duncan A., Moat N., Simonato M. (2019). Outcomes following transcatheter aortic valve replacement for degenerative stentless versus stented bioprostheses. *JACC: Cardiovascular Interventions*.

[13] Ribeiro H. B., Rodés-Cabau J., Blanke P. (2018). Incidence, predictors, and clinical outcomes of coronary obstruction following transcatheter aortic valve replacement for degenerative bioprosthetic surgical valves: insights from the VIVID registry. *European Heart Journal*.

[14] Dvir D., Leipsic J., Blanke P. (2015). Coronary obstruction in transcatheter aortic valve-in-valve implantation: preprocedural evaluation, device selection, protection, and treatment. *Circulation. Cardiovascular Interventions*.

[15] Ochiai T., Oakley L., Sekhon N. (2020). Risk of coronary obstruction due to sinus sequestration in redo transcatheter aortic valve replacement. *JACC: Cardiovascular Interventions*.

[16] Dvir D., Webb J. G., Bleiziffer S. (2014). Transcatheter aortic valve implantation in failed bioprosthetic surgical valves. *JAMA*.

[17] Bleiziffer S., Simonato M., Webb J. G. (2020). Long-term outcomes after transcatheter aortic valve implantation in failed bioprosthetic valves. *European Heart Journal*.

[18] Tarantini G., Nai Fovino L. (2020). Coronary access and TAVR-in-TAVR. *JACC: Cardiovascular Interventions*.

[19] Bieliauskas G., Wong I., Bajoras V. (2021). Patient-specific implantation technique to obtain neo-commissural alignment with self-expanding transcatheter aortic valves. *JACC: Cardiovascular Interventions*.

[20] Shivaraju A., Michel J., Frangieh A. H. (2018). Transcatheter aortic and mitral valve-in-valve implantation using the Edwards Sapien 3 heart valve. *Journal of the American Heart Association*.

[21] Webb J. G., Mack M. J., White J. M. (2017). Transcatheter aortic valve implantation within degenerated aortic surgical bioprostheses. *Journal of the American College of Cardiology*.

[22] Allen K. B., Chhatriwalla A. K., Saxon J. T. (2019). Bioprosthetic valve fracture: technical insights from a multicenter study. *The Journal of Thoracic and Cardiovascular Surgery*.

[23] Johansen P., Engholt H., Tang M., Nybo R., Rasmussen P., Nielsen-Kudsk J. E. (2017). Fracturing mechanics before valve-in-valve therapy of small aortic bioprosthetic heart valves. *EuroIntervention*.

[24] Chhatriwalla A. K., Allen K. B., Saxon J. T. (2017). Bioprosthetic valve fracture improves the hemodynamic results of valve-in-valve transcatheter aortic valve replacement. *Circulation. Cardiovascular Interventions*.

[25] Abbas A. E., Mando R., Kadri A. (2021). Comparison of transvalvular aortic mean gradients obtained by intraprocedural echocardiography and invasive measurement in balloon and self-expanding transcatheter valves. *Journal of the American Heart Association*.

[26] Komatsu I., Mackensen G. B., Aldea G. S., Reisman M., Dvir D. (2019). Bioprosthetic or native aortic scallop intentional laceration to prevent iatrogenic coronary artery obstruction. Part 2: how to perform BASILICA. *EuroIntervention*.

[27] Lederman R. J., Babaliaros V. C., Rogers T. (2019). Preventing coronary obstruction during transcatheter aortic valve replacement: from computed tomography to BASILICA. *JACC: Cardiovascular Interventions*.

[28] Khan J. M., Rogers T., Greenbaum A. B. (2020). Transcatheter electrosurgery. *Journal of the American College of Cardiology*.

[29] Khan J. M., Dvir D., Greenbaum A. B. (2018). Transcatheter laceration of aortic leaflets to prevent coronary obstruction during transcatheter aortic valve replacement. *JACC: Cardiovascular Interventions*.

[30] Khan J. M., Greenbaum A. B., Babaliaros V. C. (2019). The BASILICA trial: prospective multicenter investigation of intentional leaflet laceration to prevent TAVR coronary obstruction the BASILICA trial. *JACC: Cardiovascular Interventions*.

